# Sociodemographic characteristics associated with an eHealth system designed to reduce depressive symptoms among patients with breast or prostate cancer: a prospective study

**DOI:** 10.1192/j.eurpsy.2022.452

**Published:** 2022-09-01

**Authors:** N.G. Petros, G. Hadlaczky, S. Carletto, S.G. Martínez, B. Meyer, L. Ostacoli, M. Ottaviano, E.P. Scilingo, V. Carli

**Affiliations:** 1 Karolinska Institutet, Learning, Information, Management And Ethics, Stockholm, Sweden; 2 University of Torino, Department Of Neuroscience “rita Levi Montalcini”, Torino, Italy; 3 Universidad Politécnica de Madrid, Life Supporting Technologies, Madrid, Spain; 4 GAIA AG, Gaia Ag, Hamburg, Germany; 5 University of Turin, Department Of Clinical And Biological Sciences, Turin, Italy; 6 University of Pisa, Department Of Information Engineering, Pisa, Italy

**Keywords:** eHealth, Usability, Depression, mental health

## Abstract

**Introduction:**

Electronic health (eHealth) interventions integrate different elements of care in treating and preventing mental ill-health in patients with somatic illnesses. Identifying different sociodemographic characteristics that might be associated with higher perceived usability can help in improving the usability of these e-health interventions.

**Objectives:**

This study aimed to identify sociodemographic characteristics that might be associated with the perceived usability of the NEVERMIND e-health system, comprised of a mobile application and a sensorized shirt, developed to reduce co-morbid depressive symptoms in patients with breast or prostate cancer.

**Methods:**

The study included 129 patients with a diagnosis of breast or prostate cancer who received the NEVERMIND system. Sociodemographic data were collected at baseline. Usability outcomes included the System Usability Scale (SUS), the Mobile Application Rating Scale: user version (uMARS), and a usage index.

**Results:**

The analysis was based on 108 patients (68 breast cancer and 40 prostate cancer patients) who used the NEVERMIND system. The overall mean SUS score at 12-weeks was 73.4 with no statistical differences among different sociodemographic characteristics. The global uMARS score was 3.8, and females scored the app higher than males (β coefficient= 0.16; p=.03, 95% CI 0.02 - 0.3). Females had significant lower usage (β coefficient= -0.13; p=.04, 95% CI -0.25 to -0.01) after adjusting for other covariates.

**Conclusions:**

There was a higher favourability of the mobile application among females compared to males. However, males had significantly higher usage of the NEVERMIND system. The NEVERMIND system does not suffer from ‘digital divide’ where certain sociodemographic characteristics are more associated with higher usability.

**Disclosure:**

No significant relationships.

